# CSL112 (Apolipoprotein A‐I [Human]) Reduces the Elevation in Neutrophil‐to‐Lymphocyte Ratio Induced by Acute Myocardial Infarction

**DOI:** 10.1161/JAHA.123.033541

**Published:** 2024-05-03

**Authors:** Bronwyn A. Kingwell, Danielle Duffy, Regina Clementi, Elena Velkoska, John Feaster, C. Michael Gibson

**Affiliations:** ^1^ CSL Ltd Bio21 Institute Melbourne Victoria Australia; ^2^ CSL Behring King of Prussia PA USA; ^3^ PERFUSE Study Group Boston and Beth Israel Deaconess Medical Center Harvard Medical School Boston MA USA

**Keywords:** acute myocardial infarction, apolipoprotein A‐I, inflammation, neutrophil‐to‐lymphocyte ratio, Myocardial Infarction

After acute myocardial infarction (AMI), patients are at substantial risk of recurrent major adverse cardiovascular events (MACE), particularly during the first 90 days.^1^ Early leukocyte recruitment to the infarcted area is crucial to mediate tissue repair. Neutrophils are amongst the first cells recruited and are important mediators of the inflammatory healing response, especially during the first week.^2^ Proinflammatory neutrophils, which peak during this period, release reactive oxygen species and support phagocytic clearance of damaged cells.^2^ The intensity of neutrophil recruitment during the first week post AMI is a critical determinant of the functionality of cardiac repair. Excessive or prolonged recruitment triggers an inflammatory cascade that aggravates cellular damage,^2^ which can exacerbate myocardial injury and increase vulnerability to atherosclerotic plaque rupture.^2^ Conversely, complete neutrophil depletion adversely affects cardiac tissue remodelling^2^; thus, moderation of neutrophil recruitment in the early therapeutic window post AMI may influence the risk of experiencing recurrent MACE.

Neutrophil‐to‐lymphocyte ratio (NLR) is a clinically accessible marker of inflammation that assesses the dynamic relationship between innate and adaptive immune responses and independently predicts incident MACE in major trials.^3^ Furthermore, inflammatory inhibition with interleukin‐1β blockade, an intervention proven to reduce MACE, reduces NLR.^3^

CSL112 (apolipoprotein A‐I [human]) is a novel plasma‐derived therapy being evaluated for its ability to reduce the risk of early recurrent MACE post AMI through mechanisms that may include anti‐inflammatory actions of CSL112 on plaque stability. In a phase 2 multicenter, double‐blind study,^4^ NLR was measured to determine whether CSL112 reduces inflammation in the relevant early therapeutic window, 1 week post AMI. The study was approved by national regulatory agencies and institutional review boards or ethics committees at all participating sites. All subjects provided written informed consent before randomization. Eighty‐three patients with AMI and stage 3 chronic kidney disease were randomized 2:1 to receive 4 infusions of either 6 g CSL112 (*n*=55) or saline placebo (*n*=28) each separated by 1 week (NCT02742103). The first infusion was administered a mean of 83 hours after AMI presentation (allowed range: 12 hours–7 days). Blood samples were drawn before each infusion on days 1 (baseline), 8, 15, and 22 and on days 29 and 60. Leukocyte data were available for up to 75 participants across the 6 time points and neutrophil and lymphocyte counts were measured. Calculated NLR was non‐normally distributed, so analyses were performed on Log_e_ transformed data. Mixed‐model repeated measures analysis, with terms for treatment, visit, diabetes (yes, no), estimated glomerular filtration rate (<45, ≥45 mL/min/1.73 m^2^), baseline, and interactions between visit and the other model terms, was used to compare changes from baseline in Log_e_NLR as well as neutrophil and lymphocyte count at the 5 other time points. ANCOVA was used to assess the effect of time from index MI to first infusion on change from baseline in Log_e_NLR at day 8.

Log_e_NLR increased after AMI (between day 1 and day 8) with placebo treatment, consistent with rapid recruitment of neutrophils to the injury site within the initial days post MI. In contrast, CSL112 infusion given after the day 1 blood sample resulted in a decline in Log_e_NLR at day 8 compared with placebo (*P*=0.012; Figure [A]).

**Figure 1:**
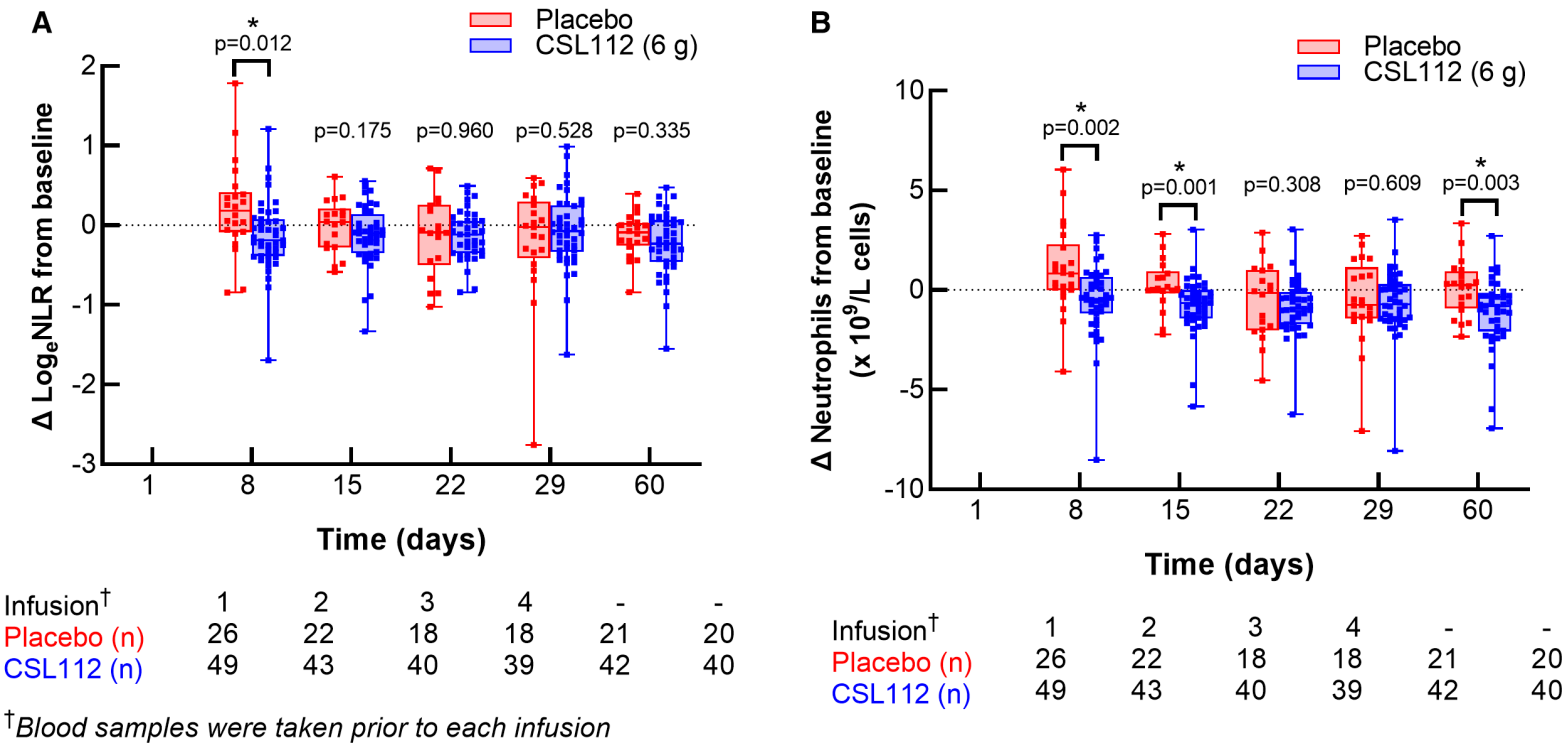
Change in Log_e_NLR (A) and change in neutrophil count from baseline (B). Data are median (line), 25th, and 75th percentile (box) with all individual points plotted (whisker). CSL112 indicates apolipoprotein A‐I (human); and NLR, neutrophil‐to‐lymphocyte ratio.

The CSL112‐induced reduction in Log_e_NLR was driven by a significantly lower neutrophil count (*P*=0.002; Figure [B]) after 8 days, with lymphocyte levels remaining stable (*P*=0.537; not shown). The reduction in neutrophils persisted and was also significant at days 15 and 60. This suggests CSL112 moderates initial neutrophil recruitment, reducing the peak of neutrophil elevation in the infarct region early after AMI. Because neutrophil recruitment is the first‐line cellular response to injury and the initiator of subsequent innate immune responses post MI, the reduction in neutrophil count induced by CSL112 would curtail the recruitment cascade of other inflammatory mediators, potentially contributing to broader anti‐inflammatory actions that may include coronary plaque stabilization. Consistent with this hypothesis, the magnitude of the effect of CSL112 to reduce NLR was greater if the timing of administration was closer to the index MI (treatment × time interaction *P*=0.008; estimated slope coefficient CSL112, 0.0052 [95% CI, 0.0003–0.0101]; placebo, −0.0046 [95% CI, −0.0098 to 0.0006]).

At a mechanistic level cholesterol efflux inhibits both neutrophil migratory function and hematopoietic stem and multipotential progenitor cell (HSPC) proliferation. Interestingly, a predecessor formulation of CSL112 (CSL111, rHDL) was shown to reduce this proliferation at 96 hours post infusion in the high‐fat diet fed ApoE^−/−^ knockout mouse model of atherosclerosis, an effect associated with reduction in circulating plasma granulocyte stimulating factor.^5^ These observations represent a plausible mechanism linking the well‐documented effects of CSL112 on cholesterol efflux elevation to anti‐inflammatory mechanisms, providing a potential explanation for the effects of CSL112 on NLR post AMI. The absence of blood sampling between days 1 and 8, when the peak of inflammation post AMI is expected, potentially contributes to an underestimation of the anti‐inflammatory effects of CSL112.

In conclusion, CSL112 attenuates post‐AMI elevation in NLR, an effect maximized when infusion occurs earlier after presentation. Whether the anti‐inflammatory effects of CSL112, in conjunction with its ability to enhance cholesterol efflux capacity, will improve cardiovascular outcomes is currently being examined.

The data that support the findings of this study are available from the corresponding author upon reasonable request.

## Sources of Funding

This work was supported by CSL Behring.

## Disclosures

Drs Kingwell and Velkoska are employees of CSL Limited (Melbourne, Australia) and Dr Duffy, Ms Clementi and Mr Feaster are employees of CSL Behring (King of Prussia, USA). Dr Gibson has received research grant support from Angel Medical Corporation, Bayer Corp, CSL Behring, Janssen Pharmaceuticals, Johnson & Johnson Corporation, and Portola Pharmaceuticals and has received modest consulting monies from Amarin Pharma, Amgen, Arena Pharmaceuticals, Bayer Corporation, Boehringer Ingelheim, Boston Clinical Research Institute, Cardiovascular Research Foundation, Chiesi, CSL Behring, Eli Lilly, Gilead Sciences, Inc, Janssen Pharmaceuticals, Johnson & Johnson Corporation, The Medicines Company, Merk & Co, Inc, Novo Nordisk, Pfizer, Pharma Mar, Portola Pharmaceuticals, Sanofi, Somahlution, St Francis Hospital, Verson Corporation, and Web MD.
